# Parasitaemia estimation and prediction of hepatocellular dysfunction among Ghanaian children with acute malaria using haemoglobin levels

**DOI:** 10.1016/j.heliyon.2021.e07445

**Published:** 2021-06-30

**Authors:** Raymond Charles Ehiem, Fareed Arthur Kow Nanse, Michael Adu-Frimpong, Felix Charles Mills-Robertson

**Affiliations:** a Department of Biochemistry and Biotechnology, College of Science, Kwame Nkrumah University of Science and Technology, Kumasi, Ghana; b Department of Applied Chemistry and Biochemistry, Faculty of Applied Sciences, C. K. Tedam University of Technology and Applied Sciences (CKT-UTAS), Navrongo, UK-0215-5321, Ghana; c Saint Patrick's Hospital, Offinso, Kumasi, Ghana

**Keywords:** Parasite density, *Plasmodium falciparum* parasitaemia, Malaria hepatopathy, Hepatocellular dysfunction, Aspartate transaminase, Alanine transaminase

## Abstract

Malaria is an important global health disease which puts individuals, particularly children, at a greater risk of mortality. *Plasmodium falciparum* is distinguished from the rest of the *Plasmodia* by its high level of parasitaemia. They infect liver cells (hepatocytes), and multiply into merozoites and rupture liver cells in the process, prior to infection of red blood cells. This study sought to estimate the extent to which *P. falciparum* parasitaemia correlates with hepatocellular dysfunction among Ghanaian children suffering from acute malaria in three malaria endemic districts in Ashanti Region and to predict liver dysfunction from the estimation of haemoglobin (HB) levels. A prospective uncontrolled before- and after study was conducted among under five years children with acute malaria (n = 300) and a control group (n = 20) within the same age brackets. The serum activities of liver enzymes such as aspartate transaminase (AST), alanine transaminase (ALT), alkaline phosphatase (ALP) and gamma glutamyl transferase (GGT) were measured in patients and control subjects. The study observed an inverse relationship between mean HB and parasitaemia (mean HB level of 10.34 ± 0.14 versus parasitaemia <10,000 parasites/μL as against 8.06 ± 0.16 versus parasitaemia ≥10,000 parasites/μL). The mean levels of AST, ALT, ALP and GGT were higher (*p* < 0.0001) in the serum of the infected children before treatment compared with post treatment. Moreover, the receiver operating characteristics (ROC) curve was applied to establish that HB level at 10.9 g/dL predicted liver dysfunction with the area under the curve (AUC) being 0.75 ± 0.03 (P < 0.0001). The parasitaemia estimation and prediction of hepatocellular dysfunction in Ghanaian children with acute malaria could be done via HB levels.

## 1 Introduction

Malaria is the single most important cause of morbidity and mortality in Ghana [1]. The disease is very prevalent in the tropics and each year approximately 300–500 million malaria infections caused by *Plasmodium falciparum* culminate in over one million deaths, wherein over 75 % occur in African children aged less than five [2, 3]. The disease can extremely progress at rapid manner and cause death within hours or days with possible consequences on vital organs including the liver [4]. It has been suggested that dysfunction of the liver may be caused by malaria infestation [5]. Although malaria hepatopathy has been considered a rare occurrence, the malady has seen an increase, particularly in endemic areas [6]. Liver compromise in people with malaria and correlates with a greater likelihood of complications and death [7, 8, 9]. *P. falciparum* malarial infection has been shown to significantly increase the serum activities of aspartate transaminase (AST), alanine transaminase (ALT) and alkaline phosphatase (ALP) in a manner that positively correlates with the parasites’ density [10]. The liver is involved in or is responsible for various haematological abnormalities due to its unique portal circulation and its metabolic (clotting factors and thrombopoietin) and immune functions. Hepatocellular dysfunction can lead to haematological abnormalities and primary diseases of haematology can in turn affect the liver and its functioning [11]. In this study, characterisation of hepatocellular dysfunction was done using ALT elevation above upper-limit of normal (ULN) [12]. Thus, levels of ALT which predominantly reflected liver injury was considered as hepatocellular dysfunction [12]. Currently, no study in Ghana had explored the power of haemoglobin (HB) levels to predict hepatocellular dysfunction in Ghanaian children less than five years. Hence, there is the need to establish the relationship between parasitaemia density and HB levels as well as the potential of the latter to predict hepatocellular dysfunction among Ghanaian children with acute malaria.

## 2 Materials and methods

### 2.1 Study design/study site

This prospective uncontrolled before-and after study was carried out between March and October, 2013 at three districts hospitals in the Ashanti Region, Ghana. These were St. Patrick's hospital at Offinso Municipal, Mankranso Government hospital at Ahafo Ano South district and St. Martin's hospital at Agroyesum, Amansie West district. The study zone was located within the forest-savannah and transitional ecological zone in Ghana. Studies in the area showed 50% prevalence of malaria parasitaemia among children less than 10 years of age (symptomatic/asymptomatic) with the transmission being perennial, and entomological inoculation rate of 269 infective bites per person per year [13].

Three hundred (300) under five years’ Ghanaian children with acute malaria were recruited for the ten-month study. The control group were twenty [14] with similar age to the matched groups. Children less than five years of age were selected because they are the most vulnerable group and represent the population mostly recruited in the majority of clinical trials of anti-malarial drugs and malarial vaccines in sub-Saharan Africa [2].

### 2.2 Ethical consideration

Written informed consent was obtained from all the caregivers of the participants. The ethical approval was granted by the local ethical committee on Human Research Publications and Ethics (CHRPE/KNUST/KATH/AP/022/13) and permission to conduct the clinical examination of subjects was obtained from the institution authorities. World Medical Association Declaration of Helsinki principles for Medical Research involving human subjects were followed to maintain the ethics.

### 2.3 Inclusion criteria

The eligible subjects were Ghanaian children aged below five years with clinical signs of malaria who were accessing health care at the outpatient department (OPD) of the three hospitals for the very first time.

### 2.4 Exclusion criteria

The exclusion criteria were any of the following; children on any form of medication and those that have viral hepatitis as well as being malnourished.

### 2.5 Questionnaire

A structure questionnaire was used to obtain information about the children's age, gender, and risk of infection, chemotherapy along with the signs and symptoms of infection.

### 2.6 Laboratory analyses

Malaria rapid diagnostic test (RDT) kits were used for field confirmation of *Plasmodium* species before samples for analyses were obtained from the study participants. Venous blood samples were taken from the children recruited on their first visit at the hospital after they met the inclusion criteria as stipulated by the study protocols at the three major malaria-endemic districts. Venous blood was taken again from the participants after treatment. Standard operating procedures (SOPs) for venipuncture developed by BD® Diagnostics (REF: CLSI H3-A6) was adhered to by the phlebotomists. Four [4] mL venous samples were collected into 0.5 mL microtainers containing ethylenediaminetetraacetic acid (K_2_EDTA-BD, USA), while 2 mL venous blood samples were also collected into clot activator tubes and the sera were later separated for storage. The samples were transported to the clinical laboratory of St. Patrick's Hospital, Offinso for analysis.

### 2.7 Laboratory procedure

#### 2.7.1 Staining of thick and thin smears

Two blood slides were prepared for each sample that came to the laboratory. Slightly modified World Health Organisation (WHO) guideline was utilised for smearing and staining technique as stated elsewhere [15]. Each slide had a measured volume of 6 μL of blood for thick thin but 2 μL was used. Ten percent (1:9 mL) for 10 min and 3% (3:97 mL) for 45–60 min fresh, working Giemsa stains were prepared using already prepared stock of Giemsa-staining solution, while working Giemsa buffer was prepared from buffer tablets. Thin and thick blood smear were stained with Giemsa after fixing the thin smear with absolute methanol. The 10% Giemsa stain was used to stain one of the two slides for preliminary slide reading to obtain results for participant management and treatment. The slide stained with 3% was given out by the slide coordinator to two competent, independent malaria microscopists. A positive smear was included with each new batch of working Giemsa stain for quality control.

#### 2.7.2 Examination of thick/thin smears

The entire smear was first screened at a low magnification (10X × 40X objective lens) to detect suitable fields with even distribution of white blood cells (WBC, 10–20 WBC/field) [13, 16]. Smears were then examined using X100 oil immersion. At least 100 high power fields were examined before a thick smear was declared negative. *P.* falciparum parasites were counted per 200 or 500 leukocytes, which were used to estimate the parasite density per microliter of blood. Thin films were examined to confirm the species identification on the thick film. A blood slide was declared positive when a concordant result was produced by two competent microscopists.

#### 2.7.3 Malarial parasite density determination

Parasite densities were recorded as a ratio of parasites to WBC in thick films. *Plasmodium* parasites were counted against 200 WBC on the thick film. Five hundred WBC were counted where less than nine parasites were counted after counting against 200 WBC. Where microscopists did the parasite counts in the thin film (against 2,000 red blood cells) as a result of heavy parasitaemia (greater or equal to 100 parasites per thick smear high power field), counted parasites were recalculated with 200 WBC. Next, parasite densities (parasite/μL of whole blood) were calculated as follows: = (Number of parasites counted/WBC counted) × WBC count/μL of participant. Also, parasite densities for all participants were calculated using assumed WBC of 5,000, 6,000, 8,000 and 10,000 μL of blood; all set by WHO to be used conveniently in facilities which lack the tools to determine patients’ absolute WBC values. Of note, 10,000 μL was adopted for the estimation of parasite density in subsequent studies because other WBC counts underestimated parasite burden during the preliminary analysis.

#### 2.7.4 Screening for viral markers: hepatitis B (HbsAg) and C (HCV)

As part of the exclusion criteria, participant qualification was based on the assumption that there was no indication of any hepatitis of viral origin. Therefore, all the samples collected were first subjected to screening tests to rule out hepatitis of viral origin.

#### 2.7.5 Antigenic confirmation using HPR-2 test kits

All the positives slides were set aside and blood samples in the corresponding tubes were used to test for the speciation double confirmation in the laboratory. The kits utilised were the *CareStart*^*TM*^ brand by ACCESSBIO. The antigen was expressed only by *P. falciparum* trophozoites [17]. Blood slides were declared positive for *P. falciparum* when a concordant result was produced by the histidine-rich protein II (HPR-2) test kits.

#### 2.7.6 Haematological analysis

The full blood counts (FBC) analysis for each participant's sample was analysed using the KX-21N Sysmex Haematology Analyzer (SYSMEX Group, France). Daily internal quality controls and scheduled external quality assessment programme were adhered to as a quality measure. Printout result for each participant was kept for documentation and statistical purposes. The parameters of interest to this study were HB and WBC counts.

#### 2.7.7 Liver function test

Samples for liver function test were obtained from EDTA anti-coagulated blood after centrifugation for 3 min at 1000 revolution per minute. The plasma was transferred into clean plain tubes. The samples were frozen at -20 °C and batches were run weekly. The analysis was performed using Selectra Pro S Chemistry (ELITech Group Company, Germany). Samples were thawed and placed in paediatric cups. Results displayed on the monitor of this equipment included; ALT, AST, ALP, gamma-glutamyl transpeptidase (GGT), total protein (TP), albumin (ALB), globulin (GLOB), direct (DBIL) and indirect bilirubin (IND) as well as total bilirubin (TBIL).

### 2.8 Statistical analysis

Data was checked for completeness and consistency and all queries resolved after double data entry using Microsoft Office Excel 2007® (Microsoft Corp., Redmond, WA, USA). Statistical analyses were performed using GraphPad Prism version 6.00 (GraphPad software, San Diego California, USA; www.graphpad.com) and Statistical Package for the Social Sciences (SPSS) version 20.00 for windows (SPSS Inc., Chicago, USA; www.spss.com). Normality of all variables was ascertained before being subjected to the statistical analyses using the D′ Agostino-Pearson procedure. Continuous variables were expressed as their mean ± SEM, while categorical variables were expressed as proportion. Comparisons of the general characteristics of children under five years with acute malaria before treatment against the treated group were performed using paired t tests, Chi-square or Fischer exact tests where appropriate. However, the general characteristics of the controls were compared with the untreated group using unpaired t-test or Chi-square or Fischer exact tests. Correlation was assessed by the Pearson's method. Areas under the Curve (AUC) for the haematological marker was measured through receiver operating characteristic (ROC) curve analysis for the diagnosis of anaemia amongst before treatment and post treatment group. The diagnostic performance characteristic in terms of sensitivity and specificity was calculated at different cut-offs for HB which showed higher AUC. One-way analysis of variance (ANOVA) followed by Turkey's multiple tests was performed to compare biochemical and haematological markers among control and treated subjects. The relationships between the various liver and haematological markers were assessed via linear regression.

## 3 Results

Table 1 presents the general characteristics of the study population. The children with acute malaria had almost the same mean age (2.41 ± 0.063 years) as their normal counterpart (2.60 ± 0.23 years). Generally, the biochemical indices such as AST, ALT, GGT, TBIL, DBIL, IND and WBC were significantly higher (*p < 0.05* for all) among children with acute malaria before treatment (BT) in comparison with the normal and post treatment (AT) groups. However, the levels of TP, ALB, GLOB and HB were significantly raised (*p < 0.05* for all) in AT groups than their BT cohorts. Likewise, ALP and TP were significantly higher (*p < 0.05* for both) among the BT subjects than the normal groups (Table 1).

**Table 1:** General characteristics of the study population.

Parameters	Normal (n = 20)	BT (n = 300)	AT (n = 300)	*P*^*1*^*Value*	*P*^*2*^*Value*
**Age (years)**	2.60 ± 0.23	2.41 ± 0.063	2.41 ± 0.06	0.4692	
**Biochemical**					
AST (U/L)	16.97 ± 1.84	50.62 ± 1.83	20.36 ± 0.64	<0.0001	<0.0001
ALT (U/L)	13.86 ± 1.70	61.22 ± 2.52	19.55 ± 0.76	<0.0001	<0.0001
AST/ALT	1.67 ± 0.26	1.14 ± 0.07	1.55 ± 0.05	0.0579	<0.0001
ALT/AST	1.00 ± 0.17	1.45 ± 0.07	1.24 ± 0.09	0.1204	0.0717
ALP (U/L)	176.0 ± 10.58	235.6 ± 5.10	229.6 ± 4.89	0.0031	0.3918
GGT (U/L)	28.89 ± 3.11	78.58 ± 3.33	35.15 ± 2.66	0.0001	<0.0001
TP (g/L)	70.51 ± 0.84	74.80 ± 0.79	81.67 ± 0.73	0.0513	<0.0001
ALB (g/L)	38.43 ± 0.82	37.11 ± 0.46	39.36 ± 0.40	0.4662	0.0002
GLOB (g/L)	31.94 ± 1.29	37.67 ± 0.71	41.69 ± 0.77	0.0389	<0.0001
TBIL (μmol/L)	7.59 ± 0.52	18.50 ± 1.26	8.67 ± 0.27	0.0263	<0.0001
DBIL (μmol/L)	2.70 ± 0.28	9.18 ± 0.68	3.89 ± 0.16	0.0145	<0.0001
IND (μmol/L)	4.90 ± 0.41	9.33 ± 0.63	4.90 ± 0.20	0.0719	<0.0001
**Haematological**					
HB (g/L)	12.60 ± 0.29	9.19 ± 0.12	12.49 ± 0.37	<0.0001	<0.0001
WBC (10^9^/L)	5.54 ± 0.27	10.76 ± 0.23	4.96 ± 0.12	<0.0001	<0.0001

Data are presented as mean ± SEM. BT = Before Treatment, AT = After Treatment, ALT = Alanine aminotransferase, AST = Aspartate aminotransferase, ALP = Alkaline phosphatase, GGT = Gamma glutamyl transferase, TP = Total Protein, ALB = Albumin, GLOB = Globulin, TBIL = Total Bilirubin, DBIL = Direct Bilirubin, IND = Indirect Bilirubin, HB = Haemoglobin level and WBC = White Blood Cells. P^1^: Comparison between Normal and BT, Reference ranges: ALT = 3–40 IU/L, AST = 5–37 IU/L, ALP = 98–279 IU/L, GGT = 10–71 IU/L, TP = 60–80 g/l, ALB = 30–55 g/L, GLOB = 20–40 g/L, TBIL = 1.70–20.0 μmol/L, DBIL = 0.00–6.80 μmol/L, IND = 1.70–17.0 μmol/L, HB = 13.0–18.0 g/dL and WBC = 2.4–10 × 109/L, P^2^: Comparison between BT and AT.

The comparison of haematological and liver markers among children with *falciparum* parasitaemia below and above 10,000 parasite/μL BT is indicated in Table 2. The number of children with parasite density less than 10, 000 μL was 138(46.0%) while those with P. *falciparum* parasitaemia of ≥10,000 μL were 162(54.0%). Most of the females had parasite density less than 10,000 μL (62.30%) and ≥10,000 μL (57.40%). The under listed liver markers; AST, ALT, ALP, GGT, TBIL, DBIL, IND and WBC significantly (*P* < 0.0001) increased in the serum of the BT cohorts when the *falciparum* parasitaemia was greater or equal to 10,000 parasites/μL. However, AST/ALT ratio and HB levels decreased significantly (*P* < 0.0001) when the *falciparum* parasitaemia was greater or equal to 10,000 parasites/μL (Table 2). On the contrary, *falciparum* parasitaemia did not have any influence on the liver proteins (TP, ALB and GLOB, Table 2).

**Table 2:** Comparison of haematological and liver markers among children with *P. falciparum* parasitaemia below and above 10,000 parasite/μL before treatment.

Parameters	Parasite Density <10,000 parasites/μL (N = 138)	Parasite Density ≥10,000 parasites/μL (N = 162)	*P Value*
**Age (years)**	2.42 ± 0.09	2.41 ± 0.09	0.9234
**Sex**			
Male (number, %)	37.70	42.60	0.0052
Female (number, %)	62.30	57.40	0.0632
**Biochemical**			
AST (IU/L)	36.44 ± 1.85	64.60 ± 2.72	< 0.0001
ALT (IU/L)	37.41 ± 2.34	84.71 ± 3.53	< 0.0001
AST/ALT	1.43 ± 0.13	0.86 ± 0.04	< 0.0001
ALT/AST	1.38 ± 0.13	1.52 ± 0.07	0.3317
ALP (IU/L)	191.8 ± 6.23	298.8 ± 6.35	< 0.0001
GGT (IU/L)	48.38 ± 3.23	108.4 ± 4.67	< 0.0001
TP (g/L)	75.42 ± 1.08	74.18 ± 1.15	0.4369
ALB (g/L)	37.58 ± 0.65	36.64 ± 0.66	0.3130
GLOB (g/L)	37.64 ± 1.01	37.69 ± 1.00	0.9700
TBIL (μmol/L)	10.77 ± 0.80	26.13 ± 2.21	< 0.0001
DBIL (μmol/L)	5.22 ± 0.50	13.08 ± 1.17	< 0.0001
IND (μmol/L)	5.55 ± 0.37	13.05 ± 1.13	< 0.0001
**Haematological**			
HB (g/L)	10.34 ± 0.14	8.06 ± 0.16	< 0.0001
WBC (10^9^/L)	7.34 ± 0.15	14.14 ± 0.19	< 0.0001

Data are presented as mean ± SEM. Reference ranges: ALT = 3–40 IU/L, AST = 5–37 IU/L, ALP = 98–279 IU/L, GGT = 10–71 IU/L, TP = 60–80 g/l, ALB = 30–55 g/L, GLOB = 20–40 g/L, TBIL = 1.70–20.0 μmol/L, DBIL = 0.00–6.80 μmol/L, IND = 1.70–17.0 μmol/L, HB = 13.0–18.0 g/dL and WBC = 2.4–10 × 10^9^/L. P: Comparison between parasite density <10,000 parasites/μL and parasite density ≥10,000 parasites/μL.

**Figure 1:**
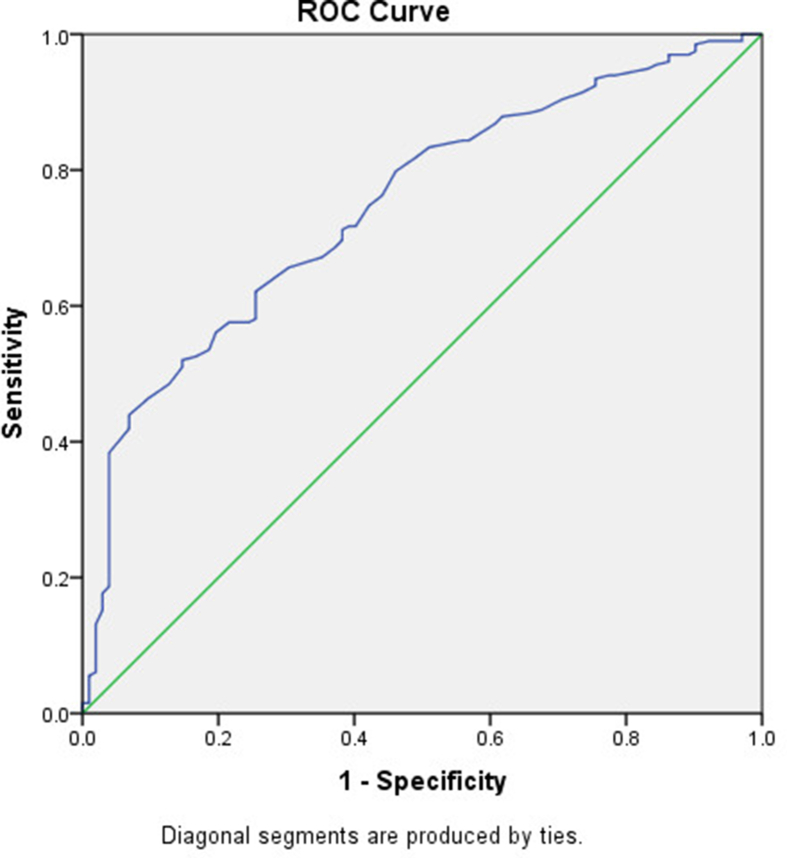
The ROC curve to detect liver dysfunction among children under five years with acute malaria before treatment.

The area under curves (AUCs) of HB which showed significant prediction of liver dysfunction is shown in Table 3 and Figure 1. The cut-off value of 10.9 g/dl for HB with the sensitivity and specificity of 86.9% and 60.8% respectively was found to detect liver dysfunction (Table 4). The Pearson's correlation coefficients of liver markers and HB for the AT and BT groups is depicted in Table 5. Notably, HB showed negative and positive correlation against AST, ALT and AST/ALT ratio respectively among the BT groups. However, HB positively associated with only ALT among the AT groups.

**Table 3:** Area under curves values of HB for liver dysfunction.

Parameters	AUC ± SEM	95% CI	*P Value*
BT			
HB (g/dl)	0.75 ± 0.03	0.69–0.80	0.0001

All values were AUC (95%CI), AUC = Area under curve, BT = Before Treatment and HB = Haemoglobin.

**Table 4:** Cut-off of HB level to predict liver dysfunction in children under five years with acute malaria before treatment.

Parameters	Cut-offs	Sensitivity	Specificity
BT			
HB (g/dl)	10.90	0.869	0.608

BT = Before Treatment, HB = Haemoglobin.

**Table 5:** Pearson's correlation coefficients of liver markers and HB for AT group (upper right-hand side and BT group (lower left-hand side).

Parameters	AST (U/l)	ALT (U/l)	AST/ALT	HB (g/l)
AST (U/l)		0.44∗∗∗	0.42∗∗∗	0.07
ALT (U/l)	0.61∗∗∗		0.79∗∗∗	0.13∗
AST/ALT	0.10	-0.37∗∗∗		-0.02
HB (g/l)	-0.36∗∗∗	-0.35∗∗∗	0.15∗∗	

BT = Before Treatment, AT = After Treatment, AST = Aspartate aminotransferase, ALT = Alanine aminotransferase. ∗Correlation is significant at 0.05 level (2-tailed), ∗∗Correlation is significant at 0.01 level (2-tailed), ∗∗∗Correlation is significant at 0.001 level (2-tailed).

**Figure 2:**
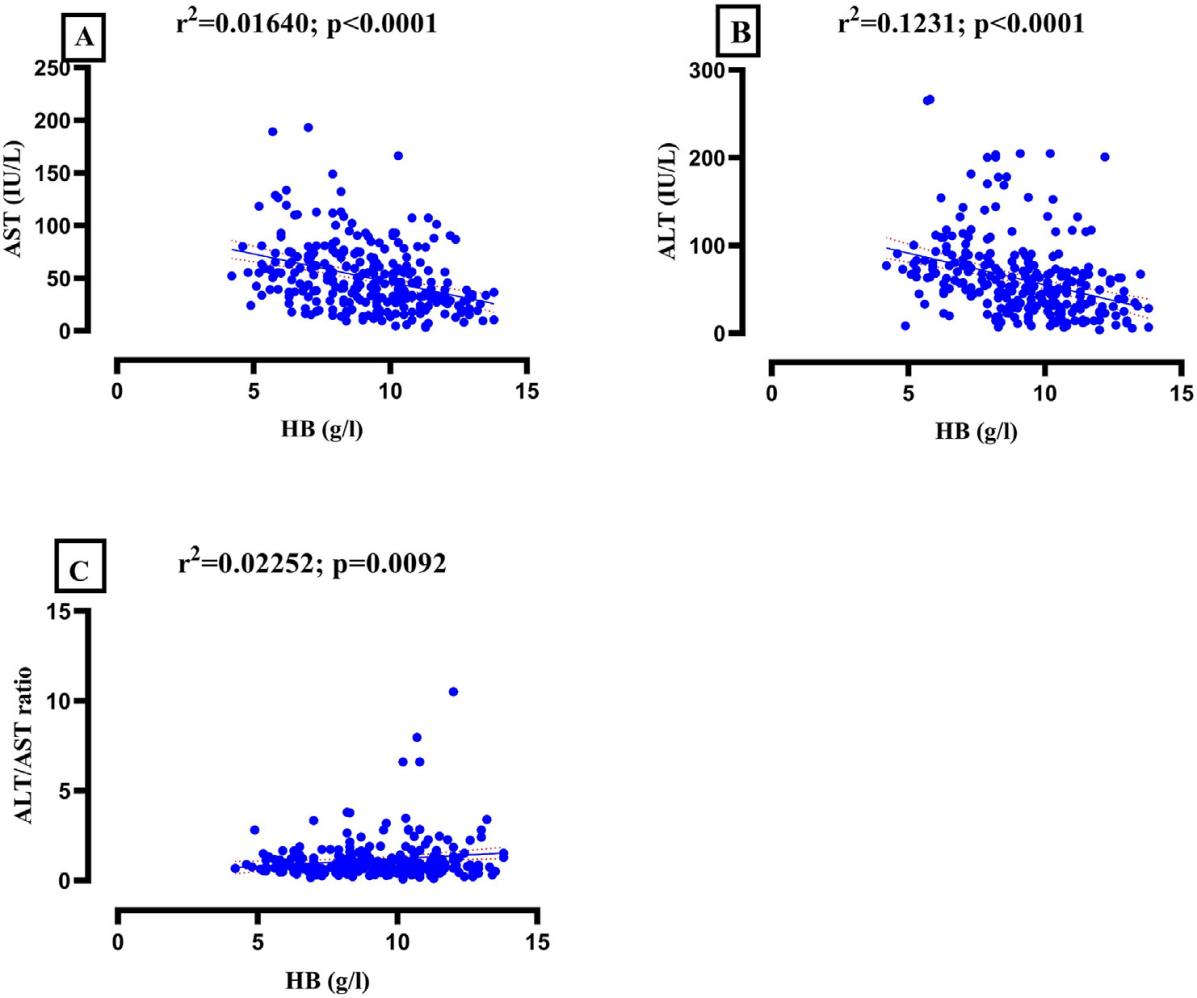
Linear regression graphs of Aspartate aminotransferase (A), Alanine aminotransferase (B) and AST/ALT ratio (C) against haemoglobin (HB) [before treatment].

**Figure 3:**
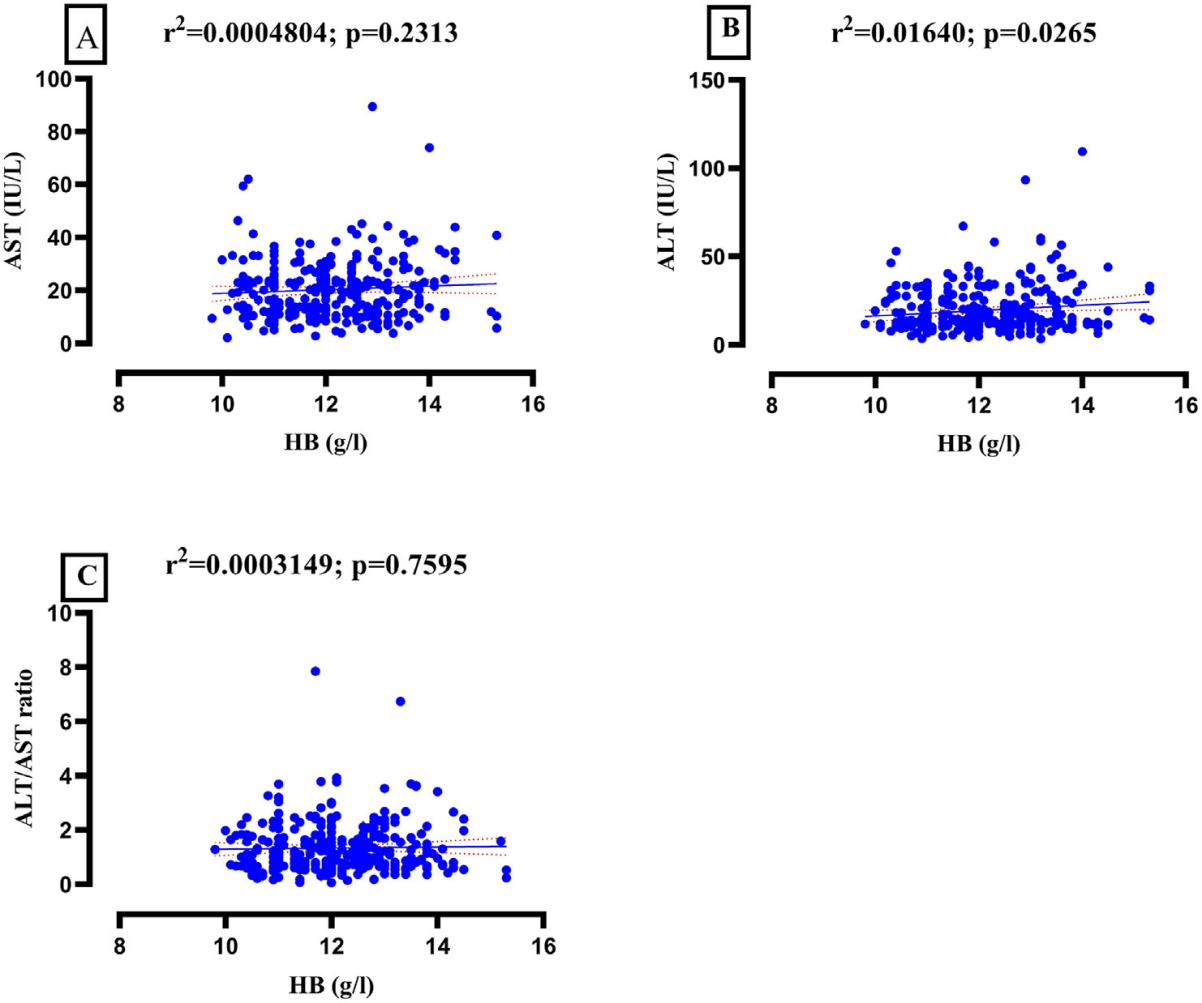
Linear regression graphs of Aspartate aminotransferase (A), Alanine aminotransferase (B) and AST/ALT ratio (C) against haemoglobin (HB) [after treatment].

The linear regression graphs of AST, ALT and AST/ALT ratio against HB for BT and AT groups are presented in Figures 2 and 3 respectively. In the BT group, HB predicted AST (r^2^ = 0.1293; p<0.0001), ALT (r^2^ = 0.1231; p<0.0001) and AST/ALT ratio (r^2^ = 0.02252; p = 0.0092). Nevertheless, HB showed linear relationship with only ALT (r^2^ = 0.01640; p = 0.0265) in the AT group.

## 4 Discussion

Acute *P. falciparum* malarial infection confers a measure of hepatic compromise [10]. This study assessed the relationship between levels of parasitaemia in blood and liver enzymes (AST, ALT, ALP and GGT) concentration in serum using newly diagnosed cases of malarial infection which is yet to be treated. The findings in the study corroborated the fact that malarial infection can cause an increase in liver enzyme concentrations in serum. This finding also agreed with earlier reports by Maegraith [18] which described the etiology of hepatic dysfunction in patients with *falciparum* malaria. Similarly, Abro *et al.* [11], observed positive correlation between parasite density and liver enzyme elevation among Pakistani children under five years. These observations could be attributed to significant perturbation in the hepatocyte membrane during the hepatic stage of the parasite's life cycle in the human host and this result in the leakage of the liver enzymes into the extracellular fluids.

The *falciparum* parasitaemia did not have any effect on the levels of liver proteins (TP, ALB and GLOB). A similar study by Kochar *et al.* [19], in India involving under five children found no appreciable elevations of the liver proteins in serum. This could be due to the fact that increased serum albumin, as well as other liver proteins are seldom encountered except in the cases of dehydration [14].

This study also posited that *P. falciparum* parasitaemia increased with elevations of serum bilirubins (TBIL, DBIL and IND) (Table 2). Ramachandran *et al.* [20], Kochar *et al.* [18], and Abro *et al.* [11], recorded similar results in their studies. Centribular damage is one of the factors involved in hepatic dysfunction in acute *falciparum* malarial infection, leading to hyperbilirubinaemia, which is a direct consequence of the impaired drainage capacity of the liver [20].

Furthermore, the study observed inverse relationship between HB concentrations and *falciparum* parasitaemia. This result agreed with a similar work by Tagelsir *et al.* [21], which found a decreased in HB concentration as parasitaemia increased. Hassan *et al.*^27^ in a related work also observed a similar linkage between parasite density and HB concentrations. Akhund *et al.* [22], established that at higher levels of parasitaemia, excessive haemolysis of parasitised red blood cells (RBCs) may lead to anaemia. Despite extensive documentation of anaemia in malaria, only mild decrease in HB was observed in this study at a parasite density ≥10,000 parasites/μL. This discrepancy may be due to the multifactorial aetiology of anaemia. Available data suggest that anaemia is categorised into mild (HB = 10.0–10.9 g/dl), moderate (HB = 7.0–9.9 g/dl) and severe (HB˂7.0 g/dl) [23]. Hence, the mild HB levels observed in this study population could reflect a lower prevalence of underlying anaemia, better nutritional status, and/or better access to treatment. Haemolysis due to plasmodium-parasitised erythrocytes becomes evident when liver function tests are conducted. Fazil *et al.* [24], posited that *P. falciparum* infection is the leading cause of malarial hepatopathy.

Because of its simplicity and routine usage, consideration was given to HB levels. The HB marker utilised in this work had relationships with the biochemical markers when compared. Overall, in the BT group, at a low HB level of 9.19 ± 0.12 g/dL, ALT, the enzyme that specifically marks for liver dysfunction was very much deranged: 61.22 ± 2.52 IU/L (Table 1). The reference range for this enzyme, according to WHO, is 3–40 IU/L. At the same low HB level, the other liver dysfunction markers were predictably deranged (Table 2). When there was a further reduction in the HB level (8.06 ± 0.16 g/dL) as a result of heavy parasitaemia, ALT again increased to 84.71 ± 3.53 IU/L (Table 2). According to Burtis *et al.* [25, 26], these parasite-induced damage to hepatocytes, accompanied by haemolysis and subsequently low HB levels, led to the leakage of parenchymal (transaminases) and membranous (alkaline phosphatase) enzymes of the liver into the circulatory system, hence the increase in liver enzymes among malaria patients. Gonzalez-Casas *et al.* [27], suggested that the frequent association of low HB level with hepatocellular dysfunction provides a rationale for examining the role of the liver in the formation and destruction of RBCs. They further opined that apart from haemolysis, a variety of mechanisms may be implicated in the development of anaemia in patients with malarial liver dysfunction.

The present study showed that HB levels could be used to predict liver dysfunction in Ghanaian under five children with acute malaria before the initiation of chemotherapy (Figure 1). Liver, spleen and the bone marrow are three parts of the body that are responsible for the production of the RBCs or the HB. The HB carries oxygen to all the parts of the body. Immature liver of children less than five years coupled with malaria infection could decrease the production of HB. This can reduce the quality of the blood cells and also cause further complications to the health of the children.

According to Mason and Graham [28], the area under the ROC curve measures discrimination, that is, the ability of the test to correctly classify those with and without hepatocellular dysfunction. The accuracy of the test depends on how well the test separates the group being tested into those with and without the condition in question. Accuracy is measured by the area under the ROC curve. An area of 1 represents a perfect test; an area of 0.5 represents a worthless test [28]. In the present study, an area of 0.75 ± 0.03 was obtained (Table 3). Fawcett [29] and Gaffikin *et al.* [30], were unanimous in their assertion that such a value is indicative of a good test.

This present study found that, regardless of gender, age and parasitaemia (based on preliminary analysis, albeit data not shown), at a HB level below 10.9 g/dl, there is 86.9% chance that liver dysfunction can be predicted (Table 4). This, of course, is plausible if factors such as viral hepatitis and chemotherapy-induced changes have been ruled out. It must be admitted that sensitivity of 86.9% and specificity of 60.8% may not be too high enough to distinguish between positive and negative outcomes, but area under the curve (AUC) of 0.75 could mean potential accuracy of the diagnostic. As an efficacious quantity of inherent validity of a diagnostic test [31], Swets [32] suggested that 0.5˂AUV˂0.7 implied less accurate prediction. This prediction is not surprising because, according to McHutchison *et al*. [33], low HB level (anaemia) of diverse etiology occurs in about 75% of patients with hepatitis c-induced liver dysfunction which was manifested by increased extrahepatic levels of liver transaminases. The comparison of the general characteristics of 20 controls with 300 patients is another limitation of this study, since inadequate sample size of controls may affect the statistical power to detect the differences between the two groups (35). Therefore, it is recommended that a similar study should be conducted in larger populations in Ghanaian children under five years in order to confirm the relationship between HB levels and hepatocellular dysfunction.

## 5 Conclusions

It was observed in this study that malarial infection could cause an increase in liver enzyme concentrations in serum of Ghanaian children under five years. Also, marked increase in plasma levels of liver enzymes and decrease in HB concentrations were observed in children with high *falciparum* parasitaemia. Low HB level could predict hepatocellular dysfunction in Ghanaian children under five years. The use of HB level to predict hepatocellular dysfunction may provide useful information which physicians cannot overlook in the management of acute malaria considering the high mortality rate of this disease in Ghana.

## Declarations

### Author contribution statement

Raymond Charles Ehiem: Performed the experiments; Wrote the paper.

Fareed Arthur Kow Nanse: Conceived and designed the experiments.

Michael Adu-Frimpong: Analysed and interpreted the data; Wrote the paper.

Felix Charles Mills-Robertson: Contributed reagents, materials, analysis tools or data.

### Funding statement

This research did not receive any specific grant from funding agencies in the public, commercial, or not-for-profit sectors.

### Data availability statement

Data will be made available on request.

### Declaration of interests statement

The authors declare no conflict of interest.

### Additional information

No additional information is available for this paper.
